# Quantitative assessment of biceps brachii muscle stiffness by using Young’s modulus–Angle curve during passive stretching in stroke patients

**DOI:** 10.3389/fphys.2023.907337

**Published:** 2023-03-08

**Authors:** Xinpei Zhang, Li Zhang, Yang Sun, Tao Li, Mouwang Zhou

**Affiliations:** ^1^ Department of Rehabilitation Medicine, Peking University Third Hospital, Beijing, China; ^2^ Department of Ultrasound, Peking University Third Hospital, Beijing, China

**Keywords:** muscle tonus, real-time shear wave elastography, modified Ashworth scale (MAS), muscle stiffness, Young’s modulus, stroke

## Abstract

**Purpose:** This study aims to use shear wave elastography (SWE) to dynamically describe the characteristics of biceps brachii muscle stiffness during passive stretching in healthy participants, investigate changes in the Young’s modulus–angle curve under various states of muscle tone in stroke patients, and develop a new method for measuring muscle tone quantitatively.

**Methods:** In total, 30 healthy volunteers and 54 stroke patients were evaluated for elbow flexor muscle tone on both sides using passive motion examination and were divided into groups based on their muscle tone status. The real-time SWE video of the biceps brachii and the Young’s modulus data were recorded during the passive straightening of the elbow. The Young’s modulus–elbow angle curves were created and fitted using an exponential model. The parameters yielded from the model were subjected to further intergroup analysis.

**Results:** The repeatability of the Young’s modulus measurement was generally good. During passive elbow extension, the Young’s modulus of the biceps brachii steadily increased as muscle tone increased, and it increased faster when the modified Ashworth scale (MAS) score got higher. The exponential model’s fitness was generally good. The curvature coefficient was significantly different between the MAS 0 group and the hypertonia groups (MAS 1, 1+, and 2 groups).

**Conclusion:** The passive elastic characteristics of the biceps brachii are consistent with the exponential model. The Young’s modulus–elbow angle curve of the biceps brachii changes in distinct ways depending on the muscle tone status. SWE can be used to quantify muscular stiffness during passive stretching as a new way of muscle tone evaluation, allowing for quantitative muscle tone evaluation and mathematical assessment of muscle mechanical properties in stroke patients.

## 1 Introduction

Muscle tone is determined not only by regulation of the nervous system but also by muscle properties such as stiffness, suppleness, and extensibility. Clinical assessments such as the modified Ashworth scale (MAS) and modified Tardieu scale (MTS) (Li et al., 2014; Meseguer-Henarejos et al., 2018) are now commonly used to assess hypertonia in conjunction with passive motion. At the same time, hypotonia evaluation methods are limited, consisting primarily of passive motion evaluation. These methods are subjective and cannot give quantitative data of a specific muscle (Bohannon and Smith, 1987; Li et al., 2014; Meseguer-Henarejos et al., 2018; Banky et al., 2019). Therefore, a muscle tone assessment method that can supplement the objective and quantitative information for the MAS is required.

The mechanical properties of the skeletal muscle are helpful in diagnosing clinical muscular disorders, assessing muscle function, and assessing rehabilitation treatment (Rosskopf et al., 2016; Gao et al., 2019). Studies on the evaluation of the mechanical properties of a single skeletal muscle, such as the passive elastic properties, are currently being conducted. The majority of research has demonstrated that measuring muscle length, elastic modulus, or torque can be used to assess the passive elastic properties and stiffness of the gastrocnemius muscle (Hoang et al., 2005; Nordez et al., 2010). The mechanical qualities of the hamstring (Le Sant et al., 2015), tibial anterior (Koo et al., 2014), and biceps brachii (Lacourpaille et al., 2013) have also been explored in several research studies. On the other hand, muscle supports human activities and is in continuous motion. Quantifying the stiffness of the skeletal muscles within the functional range is crucial in improving body functions. Muscles in different muscle tone states may have varied mechanical properties, implying that these could be used to aid muscle tone evaluation.

Shear wave elastography (SWE) is a quantitative ultrasound elastography technique that was developed in the early 1990s. It emits pulsed radiation through a probe to generate transverse shear waves in the tissue and measures the shear wave propagation speed (*c*_*s*_, m/s) quantitatively (Bamber et al., 2013; Shiina et al., 2015). The Young’s modulus (*E*, kPa) is calculated as *E =* 3*ρc*_*s*_^*2*^ (ρ is tissue density, which can be assumed to be 1,000 kg/m^3^), is used to objectively reflect the stiffness of tissues and organs, and has unique advantages in evaluating the mechanical properties of tissues *in vivo* (Koo et al., 2013; Eby et al., 2016; Lima et al., 2018). SWE has been widely used in tissues and organs such as the liver, kidneys, breast, thyroid, and prostate (Sigrist et al., 2017; Lima et al., 2018), and some studies have shown that it is also of value in musculoskeletal applications (Eby et al., 2016; Šarabon et al., 2019). Young’s modulus can be used to reflect the static muscle stiffness and muscle tone of patients with stroke, Parkinson’s disease, and cerebral palsy (Wu et al., 2017; Bilgici et al., 2018; Creze et al., 2018; Vola et al., 2018). However, muscle tone is mostly evaluated during passive movement, such as MAS and MTS. It is necessary to explore the mechanical properties of the skeletal muscle during passive motion and its relationship with muscle tone. Some studies have shown that the relationship between the shear modulus of muscle and joint angle or muscle length is fitted with an exponential model in healthy volunteers (Koo et al., 2014; Xiao et al., 2020). Therefore, the curve of modulus–angle may be used to reflect the mechanical properties of the tested muscle and establish a relationship with muscle tone. Other research, those that also apply dynamic stretching and related parameters, describe the performance of the medial gastrocnemius (Yu et al., 2022), such as slack angle (Cao et al., 2022). This may offer a new method to observe the performance of a hypertonic muscle during passive movement.

The purpose of the present study is to construct a mathematical model of the Young’s modulus–elbow angle of the biceps brachii during passive stretching and extract parameters from the model for better quantification of the mechanical properties of the passive stretching process, as well as assess the feasibility of using SWE in describing the mechanical properties during extension and explore the application potential to help muscle tone evaluation in stroke patients with hyper-/hypotonia.

## 2 Materials and methods

### 2.1 Participants

This study focused on healthy volunteers and stroke patients with unilateral hemiplegia. Stroke patients who were hospitalized in the Department of Rehabilitation Medicine of the Peking University Third Hospital from November 2019 to December 2021 were continuously recruited, as well as 30 healthy volunteers. The inclusion criteria for healthy volunteers included those 1) who were 18 years or older and 2) who could cooperate to complete the ultrasound examination and sign an informed consent form. In addition to the aforementioned criteria, the inclusion criteria for stroke patients also included those 3) who were diagnosed according to the diagnostic criteria of cerebrovascular diseases in China (version 2019) (Neurology and Chinese Stroke, 2019) and 4) whose MAS score of the elbow flexor on the hemiplegic side was ≤2. The exclusion criteria included those with 1) severe diseases of the heart, lungs, liver, and kidneys; heart function classification greater than grade I (NYHA); and symptoms and signs or examination results of respiratory failure; 2) recent (within 6 months) physical injury, fracture, or surgery; 3) a history of muscle disease; 4) limited range of motion of the elbow; 5) severe osteoporosis; 6) acute thrombosis; and 7) pregnancy.

This study was approved by the Medical Science Ethics Committee of the Peking University Third Hospital (No. IRB00006761-M2019417). All participants signed an informed consent form before the examination.

### 2.2 Method

#### 2.2.1 Basic information collection and grouping

This was a cross-sectional study, and the basic information of our participants was collected, such as age, sex, height, body mass, body mass index (BMI), and dominant side. The basic data such as the type of disease and time of onset of the patients were also collected. The unilateral upper limbs of all participants were grouped according to the muscle tone status, such as the hypotonia group, MAS 0 group, MAS 1 group, MAS 1+ group, MAS 2 group, healthy group (HG), and unaffected side of patient group (UG). Due to the differences in age and sex of our participants, the data of the healthy group (HG) and unaffected side of the patient group (UG) were combined as the control group. According to the age of the participants, the control group was divided into youth, <45 years; middle-aged, 45–60 years; and elderly, >60 years.

#### 2.2.2 Muscle tone evaluation of elbow flexor

Before the participants underwent an ultrasound examination, the same rehabilitation physician used passive stretching and MAS to assess the muscle tone of the participants’ elbow flexor on both sides of the upper limbs.

Hypotonia: in the process of passive joint movement, the resistance disappears. The limb falls rapidly when it is placed in the antigravity limb position, and the specified limb position cannot be maintained.

MAS 0: no tonus increase.

MAS 1: muscle tension slightly increased: the presence of a catch-and-release feeling at the end of the range of motion or a slight tonus increase in character with minimal resistance.

MAS 1+: a slight increase in muscle tension: there is a slight increase in the muscle tone observed through minimal resistance throughout less than half of the joint range of motion.

MAS 2: muscle tension increases significantly: the muscle tone is increased in most of the range of motion of the whole joint, but the joints can be moved easily.

#### 2.2.3 Ultrasonography

**FIGURE 1:**
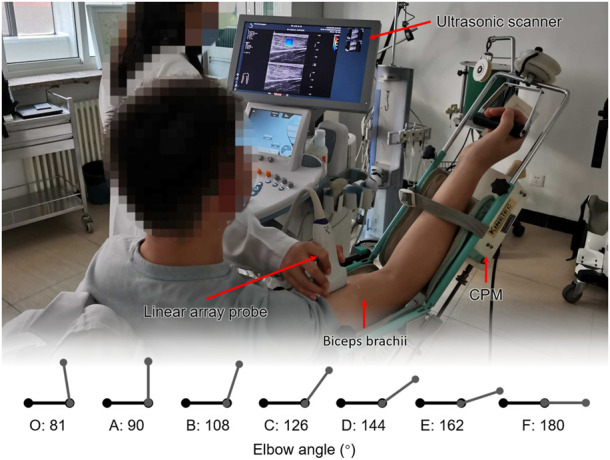
Participant and the experimental setting. Each participant took a sitting position. Their shoulder abducted 5°–10°, flexed 90° on the test side, and their upper limb was placed on the continuous passive motion (CPM) exerciser. The study started from the elbow flexion at 100°, that is, 80° straightening (the angle between the forearm and the upper arm was 80°) and ended at the extension position.

A participant and the experimental setting are shown in Figure 1. Each participant took a sitting position. Their shoulder abducted 5°–10°, flexed 90° on the test side, and their upper limb was placed on the continuous passive motion (CPM) exerciser (6080 Elbow CPM, Kinetec France). The CPM rotation axis was ensured to match the participant’s upper limbs and was then fixed. The study started from the elbow flexion at 100°, that is, 80° straightening (the angle between the forearm and the upper arm was 80°) and ended at the extension position. The movement speed of the CPM was 2.25°/s. The participants were asked to stay as relaxed as possible, and the bilateral upper limbs of each participant were tested. The patients were asked to take the first measurements on the healthy side to familiarize themselves with the procedure. The healthy participants were randomized to bilateral measurements. Each participant underwent one cycle of pretreatment for each upper limb. After the participants were familiar with the procedure and fully relaxed, the formal measurement and data collection began. Two formal measurements were taken on each upper limb. Two consecutive trials on the same side were performed and then changed to the contralateral side. The participants were told to rest for 2 min between each trial. Eight healthy volunteers were randomly selected to assess the repeatability of the measurements. The result of the better trial was used in further curve fitting and following statistical analysis.

An Aixplorer ultrasound scanner (Aixplorer, Acoustics France), coupled with a linear array probe (4–15 MHz) was used in our study. The appropriate transducer alignment was confirmed by tracing several fascicles without interruption across the B-mode image. The sonographer held the ultrasound probe in hand and placed the probe on the belly of the participant’s biceps. Throughout the scanning, care was taken to not press and deform the muscle. The acquisition frequency of elastography was 1 Hz. The real-time shear wave elastography imaging videos were collected to evaluate the changes of the Young’s modulus. All gray-scale images, elastograms, and video data were stored in the hard disk of the ultrasound equipment as original data. All ultrasound examinations were performed by the same sonographer. The average value of the Young’s modulus (kPa) in the region of interest (ROI) was obtained manually by the sonographer, and the records were checked by two researchers.

The initial Young’s modulus value for the extended position of the elbow was recorded as *E*_*180*_. Instantaneous Young’s modulus was measured every 1 s and recorded as *E*_*X*_, where X referred to the elbow angle, and the gap of X was 2.25°. The video was inverted, and the Young’s modulus was recorded frame by frame from the extended position. In total, 45 frames were checked and recorded until it reached *E*_*81*_.

Seven elbow positions were selected: O-start point, A-90°, B-108°, C-126°, D-144°, E-162°, and F-180°. Positions A–G should be approximately 3/8 joint range of motion (ROM), 1/2 ROM, 5/8 ROM, 3/4 ROM, 7/8 ROM, and full ROM (1/8 ROM and 1/4 ROM were not included in the study for the reason that ultrasound was not available). The statistical analysis was mainly conducted at these six positions.

#### 2.2.4 Construction of Young’s modulus–elbow angle curve and parameter acquisition

The scatter diagram of Young’s modulus–elbow angle was drawn for each set of data, and the elbow angle and Young’s modulus were estimated and fitted by using the IBM SPSS 22.0 software. An exponential model was used to curve-fit the data, as shown in Eq. 1:

$$E_{X}=\beta \exp\left(\alpha X\right),$$ (1)

where *E*_*X*_ is the instantaneous Young’s modulus, X is the elbow angle, *α* is the curvature coefficient of the curve, which represents the change rate of Young’s modulus of the long head of the biceps brachii during elbow extension (curvature of the curve in the coordinate axis), and *β* is the constant coefficient of the curve.

#### 2.2.5 Statistical analysis

The comparison of the count data was performed by the Chi-square test, and the comparison of the mean between groups was performed by the independent sample *t*-test. Reliability was analyzed with the two-way random intraclass correlation coefficient (ICC). Mixed-model ANOVA was used to evaluate the differences in the distribution of the Young’s modulus curve of the biceps brachii in patients with different muscle tone statuses. The Shapiro–Wilk test and Q-Q plot were used to evaluate normal distribution. If the data met the normal distribution, the two-way ANOVA was used to evaluate the differences between groups, otherwise the Mann–Whitney *U* test or Kruskal–Wallis *H* test was used. The SPSS 22.0 software was used to conduct all the statistical analyses, and a confidence level of 0.05 was chosen for all statistical tests.

## 3 Results

### 3.1 Basic information of participants

A total of 30 healthy volunteers and 54 stroke patients were recruited in this study. The basic information of the participants in each group is summarized in Table 1. Among all stroke patients, there were 28 patients with left hemiplegia and 26 patients with right hemiplegia; 9 patients with cerebral hemorrhage, and the rest 45 patients were diagnosed with cerebral infarction. The average disease duration of the patients was 57.6 ± 6.37 days. The age of the healthy volunteers was significantly younger than that of stroke patients (*p* < 0.001). The age had no statistical impact on the SWE measurement results of each position (*p* > 0.05, Supplementary Table S1). At the position of O-81° and A-90°, the Young’s moduli were lower in women than in men (*p* < 0.05, Supplementary Table S2).

**TABLE 1:** Basic information of participants in each group.

	HG (*n* = 60)	UG (*n* = 54)	Hypotonia group (*n* = 4)	MAS 0 group (*n* = 9)	MAS 1 group (*n* = 25)	MAS 1+ group (*n* = 12)	MAS 2 group (*n* = 4)	χ^2^/F value	*p*-value
Sex								7.678^a^ (b)	0.243^a^
Male	32 (53.3%)	38 (70.4%)	3 (75.0%)	8 (88.9%)	18 (72.0%)	7 (58.3%)	2 (50.0%)		
Female	28 (46.7%)	16 (29.6%)	1 (25.0%)	1 (28.0%)	7 (28.0%)	5 (41.7%)	2 (50.0%)		
Age, mean ± SD (years)	32.4 ± 8.5	59.7 ± 11.4	62.8 ± 4.6	58.9 ± 15.0	60.8 ± 10.5	57.3 ± 13.2	58.0 ± 9.3	43.390	<0.001^b^
BMI, mean ± SD (kg/m^2^)	23.3 ± 3.5	24.1 ± 3.1	22.3 ± 2.3	23.9 ± 3.4	24.7 ± 3.3	24.2 ± 2.5	24.4 ± 4.0	0.852	0.532^b^

The average age of the healthy group was significantly lower than that of the stroke patients. There was no statistically significant difference in sex and body mass index between the groups. Abbreviations: HG, healthy volunteer group; UG, unaffected side of patient group; MAS, modified Ashworth scale; BMI, body mass index; SD, standard deviation. a, using chi-square test; b, using two-way ANOVA. The average age of the healthy participants was younger than that of the patients in each group.

### 3.2 Repeatability and Young’s modulus–elbow angle curve

**FIGURE 2:**
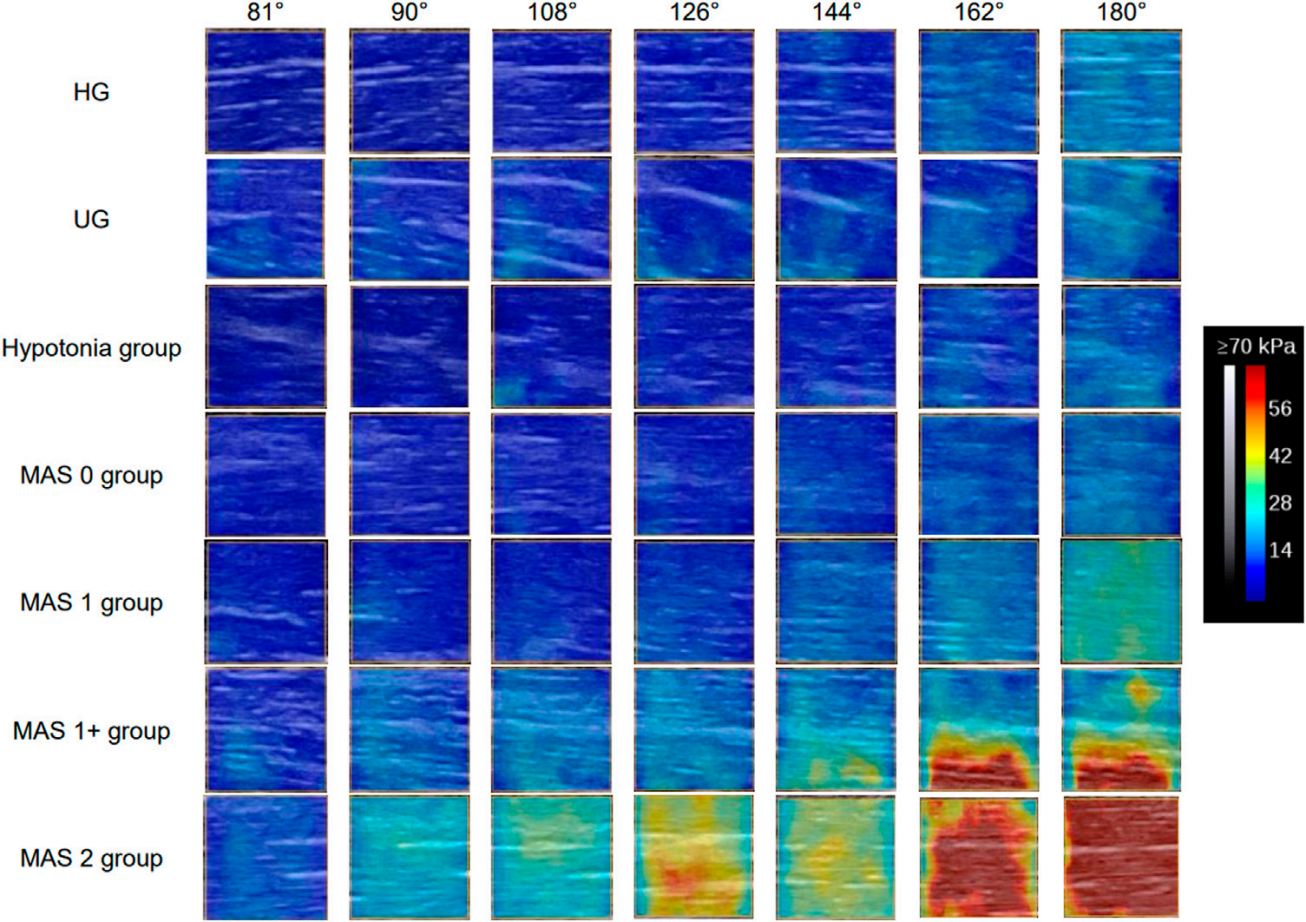
Typical SWE images of each observation positions of the participants in each group. The Young’s modulus of the biceps brachii increases continuously during the elbow straightening. With the increase of muscle tone, the degree of increase of the Young’s modulus becomes more and more obvious, and the position at which the increase of the Young’s modulus value begins to appear is earlier. Abbreviations: HG, healthy volunteer group; MAS, modified Ashworth scale; UG, unaffected side of patients.

**FIGURE 3:**
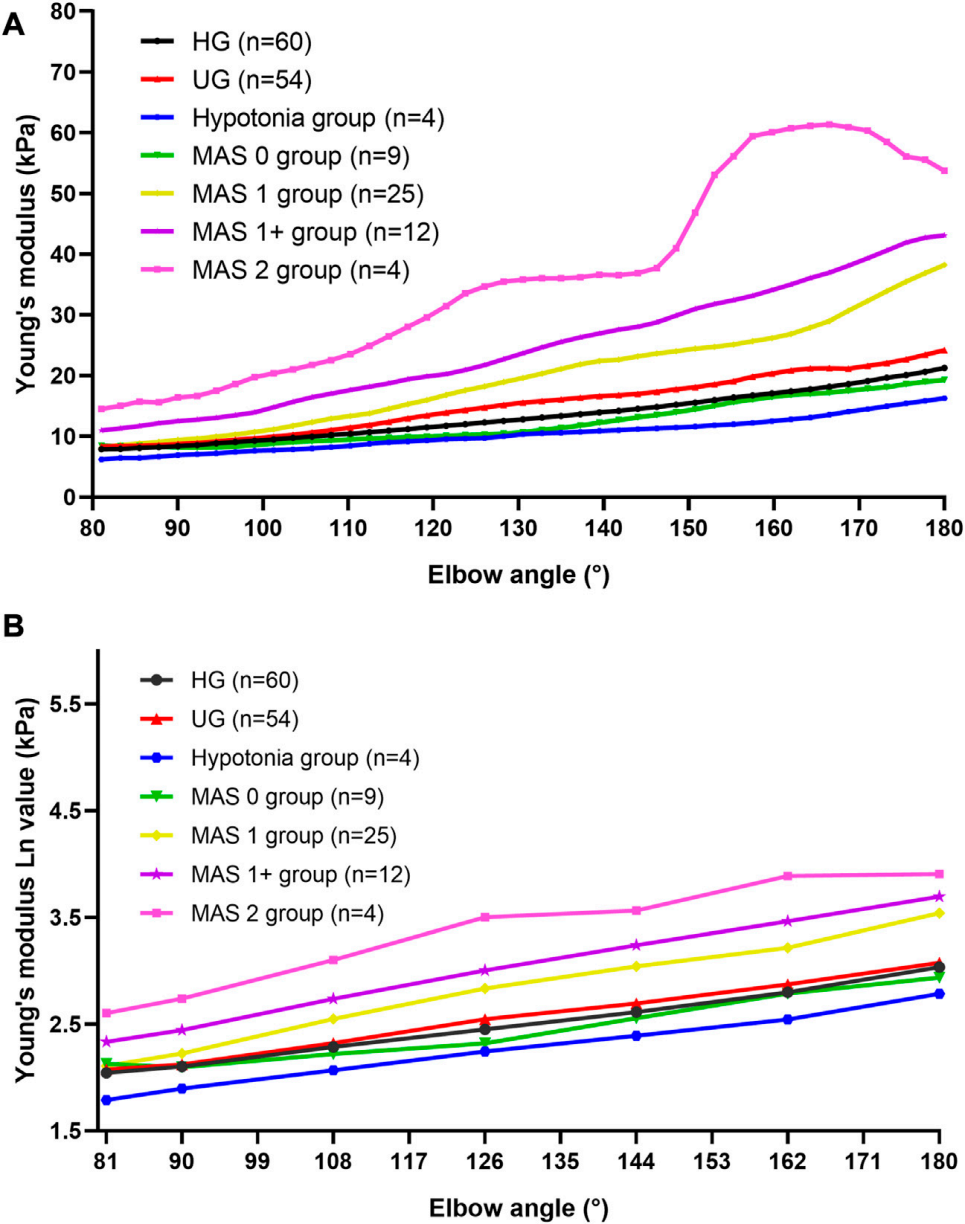
Average Young’s modulus–angle curves and Ln (*E_X_*)-angle plots of each group. **(A)** Average Young’s modulus–angle curves of each group. The Young’s modulus of the biceps brachii gradually increased as the elbow was stretching. In patients with higher MAS, the increase of the Young’s modulus occurred earlier and more obviously. **(B)** Ln (*E_X_*)-angle plots of each group. Abbreviations: HG, healthy volunteer group; MAS, modified Ashworth scale; UG, unaffected side of patients.

The Young’s modulus data in this study were not normally distributed according to the results of the Shapiro–Wilk test, so the Young’s modulus of each group was represented by the median and quartile and is summarized in Table 2. The typical SWE ultrasound images of the participants in each group are shown in Figure 2. The repeatability of the Young’s modulus measurements was generally good, with an ICC range from 0.740 to 0.872 (Table 3). We took the average of all Young’s moduli in each group and plotted each joint angle and the corresponding average of the Young’s modulus of the biceps brachii into a curve, as shown in Figure 3A. With the elbow straightened, the Young’s modulus of the biceps brachii gradually increased. In patients with higher MAS, the increase of Young’s modulus occurred earlier and more obviously.

**TABLE 2:** Young’s modulus value of each position in each group.

Position	HG (*n* = 60)	UG (*n* = 54)	Hypotonia group (*n* = 4)	MAS 0 group (*n* = 9)	MAS 1 group (*n* = 25)	MAS 1+ group (*n* = 12)	MAS 2 group (*n* = 4)
O: 81°, start point	7.550 (6.725, 8.850)	8.000 (6.825, 9.425)	7.700 (5.550, 9.325)	8.600 (6.750, 9.300)	8.100 (6.550, 10.400)	10.050 (7.625, 13.000)	11.550 (10.300, 21.725)
A: 90°, 3/8 ROM	8.100 (6.800, 9.575)	8.200 (6.975, 9.925)	8.150 (6.400, 9.900)	7.900 (6.950, 8.600)	8.600 (7.800, 11.200)	10.450 (7.950, 18.650)	13.750 (11.750, 23.700)
B: 108°, 1/2 ROM	9.700 (8.300, 11.375)	9.500 (7.875, 12.225)	9.350 (7.475, 11.375)	8.700 (7.500, 10.350)	12.300 (10.300, 17.250)	15.100 (10.100, 21.600)	21.650 (18.850, 27.150)
C: 126°, 5/8 ROM	11.300 (9.425, 13.075)	10.950 (8.825, 18.125)	11.000 (8.575, 12.750)	9.800 (8.200, 11.550)	17.200 (12.950, 25.050)	21.200 (14.650, 27.700)	34.550 (24.150, 45.325)
D: 144°, 3/4 ROM	13.000 (11.200, 14.600)	12.950 (10.200, 19.775)	12.500 (10.125, 14.500)	13.300 (9.800, 14.000)	21.400 (15.700, 26.800)	28.500 (16.950, 37.625)	35.000 (25.800, 49.825)
E: 162°, 7/8 ROM	15.200 (13.425, 18.325)	16.050 (13.475, 23.300)	13.700 (12.475, 15.225)	15.800 (12.300, 19.100)	26.400 (17.750, 31.850)	32.650 (21.275, 50.825)	41.400 (27.550, 113.225)
F: 180°, end point	19.700 (16.750, 23.825)	19.850 (16.675, 27.825)	16.850 (15.500, 18.950)	19.100 (14.800, 23.350)	36.300 (24.300, 42.400)	42.350 (29.550, 56.675)	44.000 (36.125, 81.050)

Young’s modulus for each group at seven observation positions: O and A–G. With the elbow straightened, the Young’s modulus of the biceps brachii gradually increased. The Young’s modulus data in the table are shown as median (lower quartile and upper quartile) (kPa). Abbreviations: HG, healthy volunteer group; UG, unaffected side of patient group; MAS, modified Ashworth scale; ROM, range of motion.

**TABLE 3:** Repeatability of Young’s modulus measurement at each elbow position.

Position	First measurement	Second measurement	ICC (confidence interval)
O: 81°, start point	7.400 (5.925, 8.300)	7.400 (7.100, 8.625)	0.872 (0.680, 0.953)
A: 90°, 3/8 ROM	7.650 (6.200, 9.350)	7.850 (6.850, 8.475)	0.775 (0.469, 0.915)
B: 108°, 1/2 ROM	9.150 (7.225, 10.925)	9.300 (8.150, 10.100)	0.773 (0.467, 0.914)
C: 126°, 5/8 ROM	10.700 (8.675, 11.800)	10.800 (9.325, 11.975)	0.843 (0.610, 0.942)
D: 144°, 3/4 ROM	12.150 (10.075, 13.000)	12.250 (10.850, 14.775)	0.767 (0.458, 0.911)
E: 162°, 7/8 ROM	14.200 (12.625, 14.900)	14.800 (13.450, 17.475)	0.740 (0.253, 0.911)
F: 180°, end point	18.850 (16.525, 20.450)	19.550 (16.975, 22.550)	0.756 (0.442, 0.906)

The repeatability of the measurement was evaluated in eight healthy subjects, and the results were generally good. The Young’s modulus data in the table are shown as median (lower quartile and upper quartile) (kPa). Abbreviations: ICC, interclass correlation coefficient factor; ROM, range of motion.

The logarithmic transformation of the Young’s modulus values of each group at each position was performed, and the transformed statistical data [Ln(*E*_*X*_)] met the normal distribution and the statistical requirements of the repeated measurement analysis of variance. Figure 3B shows the Ln(*E*_*X*_) at six locations. The results of the mixed-model ANOVA suggest that there are statistical differences in the distribution of the curves of each group [F (15.814, 363.731) = 6.609, *p* < 0.001]. That is, the higher the muscle tone, the higher is the Young’s modulus of the biceps brachii and the faster the Young’s modulus increases during the passive extension of the elbow.

### 3.3 Parameter of Young’s modulus curve and MAS classification

We drew scatter plots of the Young’s modulus–elbow angle curves of all the participants, and an exponential model was used to fit the data. Each curve could obtain two parameters: curvature coefficient *α* and constant coefficient *β*.

**FIGURE 4:**
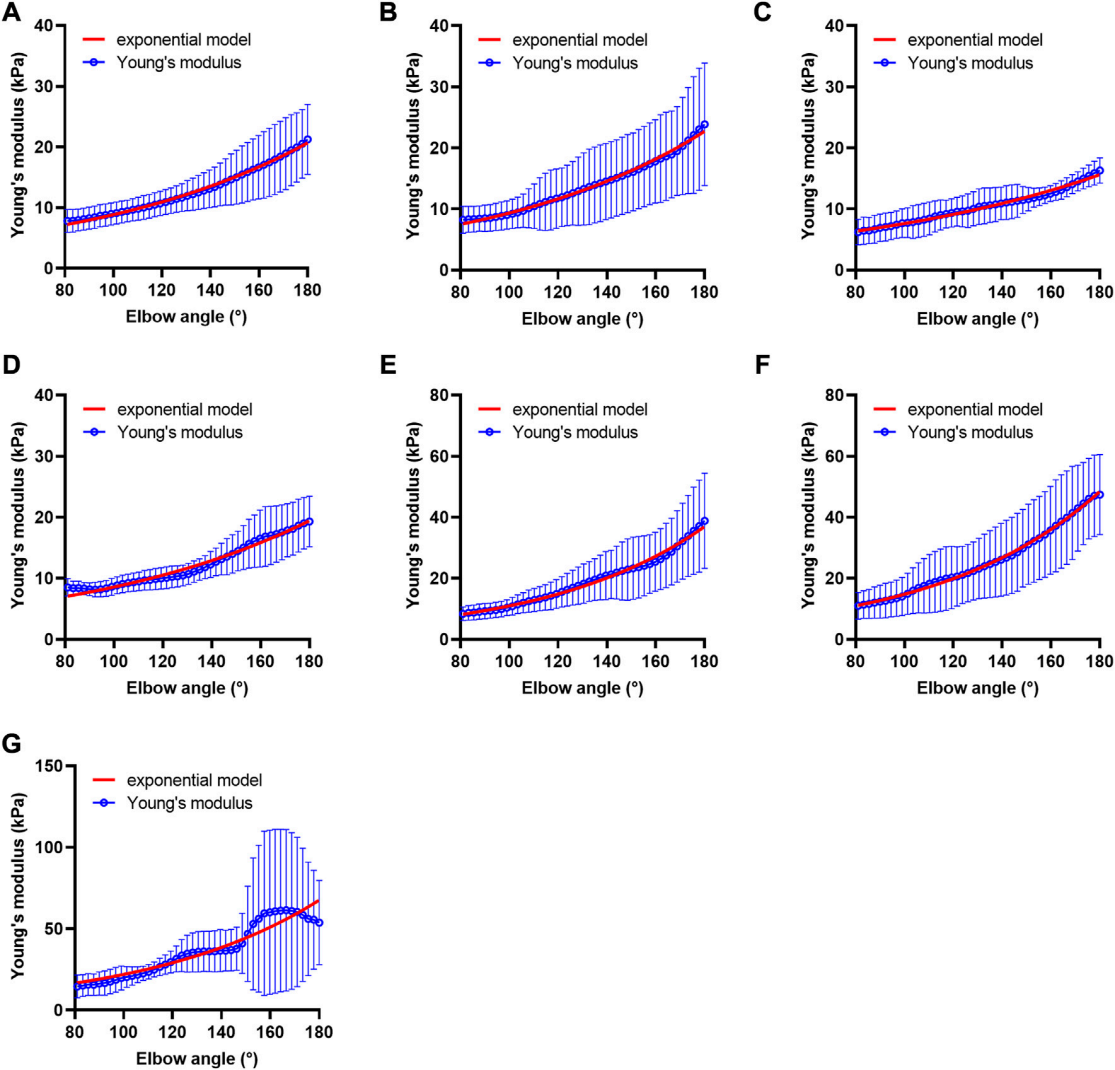
Fitted curves of Young’s modulus of each group. **(A)** Fitted curve of the healthy volunteer group, *n* = 52, **(B)** fitted curve of the unaffected side of the patient group, *n* = 43, **(C)** fitted curve of the hypotonia group, *n* = 4, **(D)** fitted curve of the MAS 0 group, *n* = 9, **(E)** fitted curve of the MAS 1 group, *n* = 22, **(F)** fitted curve of the MAS 1+ group, *n* = 10, and **(G)** fitted curve of the MAS 2 group, *n* = 4. Alpha value of MAS 1, MAS 1+, and MAS 2 groups is higher than that of HG, UG, hypotonia group, and MAS 0 group. Abbreviations: HG, healthy volunteer group; MAS, modified Ashworth scale; UG, unaffected side of patient group.

The fitness of most participants’ data to the exponential model was good. The adjusted *R* ^2^ of 59.5% of the data could reach more than 0.9, and 85.7% of them could reach more than 0.7. The data of participants with adjusted *R* ^2^ below 0.7 were considered poor fitting and were not included in the subsequent statistical analysis (data of 24 cases). Among these, 8 cases (13.3%) were in the HG, 11 cases (20.4%) were in the UG, 3 cases (12.0%) were in the MAS 1 group, and the rest 2 cases (16.7%) were in the MAS 1+ group. The overall trends of the Young’s modulus data and fitted exponential function curve of each group are shown in Figure 4.

In 144 cases with good fit, the curvature coefficient *α* and constant coefficient *β* of each group are summarized in Table 4, as well as the comparison results between the groups are shown. The results of the two-way ANOVA further suggests that there is a statistical difference of *α* among the groups (F = 6.308, *p* < 0.001, *η* ^2^ = 0.216). There was no significant statistical difference among the four groups of HG, UG, hypotonia, and MAS 0, as well as among the three groups of MAS 1 group, MAS 1 + group, and MAS 2 group (*p* > 0.05). However, there was a significant difference between the former four and the latter three groups (*p* < 0.05) (except for UG, hypotonia group, and MAS 2 group, *p* > 0.05). At present, the curvature coefficient *α* may be used to characterize whether the muscle tone is increased.

**TABLE 4:** Curvature coefficient *α* and constant coefficient *β* of each group.

Parameter	Group	N	Mean values ± standard deviation	Confidence interval
Curvature coefficient *α*	HG	52	0.0102 ± 0.0037	0.0092, 0.0112
	UG	43	0.0107 ± 0.0036	0.0095, 0.0118
	Hypotonia group	4	0.0095 ± 0.0029	0.0049, 0.0141
	MAS 0 group	9	0.0093 ± 0.0035	0.0067, 0.0120
	MAS 1 group	22	0.0147 ± 0.0049^cfgl^	0.0126, 0.0169
	MAS 1+ group	10	0.0152 ± 0.0041^cfgk^	0.0123, 0.0181
	MAS 2 group	4	0.0143 ± 0.0046^aj^	0.0069, 0.0216
Constant coefficient *β*	HG	52	3.4684 ± 1.5467	3.0378, 3.8990
	UG	43	3.2834 ± 1.3426	2.8691, 3.6977
	Hypotonia group	4	3.2448 ± 1.8721	0.2658, 6.2237
	MAS 0 group	9	3.6327 ± 1.2596	2.6644, 4.6009
	MAS 1 group	22	2.7801 ± 1.4728	2.1271, 3.4331
	MAS 1+ group	10	3.6917 ± 2.6256	1.8135, 5.5699
	MAS 2 group	4	4.8730 ± 1.0941	3.1320, 6.6140

In 144 well-fitted Young’s modulus–elbow curves, there was a statistically significant difference in the *α* value of each group. The *α* of HG, UG, hypotonia group, and MAS 0 group was lower than that of MAS 1 group, MAS 1+ group, and MAS 2 group. Abbreviations: HG, healthy volunteer group; UG, unaffected side of patient group; MAS, modified Ashworth scale. a, compared with HG, *p* < 0.05; b, compared with HG, *p* < 0.01; c, compared with HG, *p* < 0.001; d, compared with UG, *p* < 0.05; e, compared with UG, *p* < 0.01; f, compared with UG, *p* < 0.001; g, compared with hypotonia group, *p* < 0.05; h, compared with hypotonia group, *p* < 0.01; i, compared with hypotonia group, *p* < 0.001; j, compared with MAS 0 group, *p* < 0.05; k, compared with MAS 0 group, *p* < 0.01; l, compared with MAS 0 group, *p* < 0.001.

And for constant coefficients *β*, no statistical difference between the groups was found (F = 1.321, *p* = 0.252).

## 4 Discussion

The present study was designed to explore the response of the biceps brachii in healthy volunteers during passive stretching *in vivo* by Young’s modulus and find out the parameters that can be used to characterize the biomechanical properties of the biceps brachii. The parameters were also discussed in patients with elbow flexor muscle tone changes to further evaluate the potential of a quantitative assessment of the muscle tone in the future. We found that the Young’s modulus of the biceps brachii in healthy volunteers and patients changed exponentially during passive stretching. The curvature coefficient *α* of the curve of the elbow flexor might be used to distinguish between normal people and patients with hypertension.

The shear modulus or Young’s modulus measured by SWE quantifies tissue stiffness because shear waves travel faster in stiffer tissues. When compared with healthy muscle cells, spastic muscle cells have shorter resting sarcomeres and higher elastic moduli in patients with cerebral palsy (Fridén and Lieber, 2003), which indicates that muscle stiffness may reflect changes in spasticity-related tissue properties. Previous studies have found that the static Young’s modulus of a muscle at a certain body position is related to the MAS scores, that is, the higher the MAS score, the higher is the stiffness of the measured muscle, and the higher will be the Young’s modulus value measured by SWE (Wu et al., 2017; Vola et al., 2018).

However, in clinical practice, the muscle tone of the patient’s limbs during movement deserves more attention. Commonly used clinical scales such as the MAS also focus on the performance of limbs during movement. For this point, this study mainly explored the changing characteristics and mechanical properties of muscle stiffness during movement. The speed adopted in this study is 2.25°/s, which is believed to be slow enough that it cannot elicit the stretch reflex of the limbs (Eby et al., 2016; Leng et al., 2019). Therefore, this study mainly explored the mechanical properties of the muscle itself during passive stretching. On this basis, future studies could introduce higher movement speeds and possibly the use the coefficient *α* to assist in the muscle tone assessment.

Researchers have already found that there was a good exponential correlation between passive tension and length in isolated animal experiments (Herbert and Balnave, 1993; Herbert and Crosbie, 1997). Hoang et al. (2005) further verified this exponential model in the study of the gastrocnemius muscle *in vivo*. They found that there was a piecewise exponential model relationship between muscle length and tension generated by the gastrocnemius muscle during passive stretching, and nine parameters have been proposed to characterize the biomechanical properties of the gastrocnemius muscle according to their model. This method has been continuously updated in follow-up studies. Nordez et al. (2010) optimized the piecewise exponential model of passive tension and gastrocnemius muscle length to describe the biomechanical characteristics of the gastrocnemius muscle through two model parameters that included parameter *α*, which was used to explore the change characteristics of gastrocnemius muscle stiffness. The curvature coefficient *α* proposed in this study is similar to this, which can be used to characterize the ability of the skeletal muscle to resist deformation under passive stretching. In the group of participants with hypertonia (MAS 1, 1+, and 2 groups), *α* was higher than it was for the four groups without hypertonia (HG, UG, hypotonia group, and MAS 0 group).

The Young’s modulus–elbow angle curve increased more significantly when the elbow extension angle increased, indicating that the muscles in stroke patients with hypertonia have increased muscle stiffness and are difficult to adapt to muscle deformation caused by the stretching process. This increase in muscle stiffness and resistance is currently thought to be mainly due to the changes in the structure of the muscle fibers, such as shortening of muscle bundle length, increase in myometrial and myofibrils in collagen, abnormal accumulation of extracellular matrix and titin, and increase in fat content (Lieber et al., 2003), as well as changes of resting sarcomeres length that is mentioned above (Fridén and Lieber, 2003). These changes may jointly cause an increase in muscle stiffness, which is manifested by an increase in the Young’s modulus. More basic and clinical studies are required for further verification. Resistance in the later stage of the joint range of motion is also consistent with the description of the MAS.

As a pilot study, this study still has many limitations: 1) the sex difference of the participants may have an impact on the measurement results of this study (Eby et al., 2015; Miyamoto et al., 2018). 2) SWE is a measurement tool with high static test–retest reliability (Mathevon et al., 2018; Mendes et al., 2018). The reliability of the dynamic measurement method during the extension had also been confirmed in previous studies: the ICC measured by repeated SWE can reach 0.71–0.94 in the hamstrings (Le Sant et al., 2015) and 0.75–0.97 in the biceps brachii (Eby et al., 2016). In the present study, the ICC ranged from 0.740 to 0.872; however, the measurement reliability still has to be further evaluated in a larger sample size. 3) The straightening speed used in the study was slow and did not cause the stretch reflex of the limbs. 4) As MAS score 3 is characterized by passive movement difficulties, MAS score 4 is characterized by stiffness and inability to complete passive activities. Therefore, patients with MAS scores 3 and 4 were not included in the study for the reason that it would be difficult for them to complete the protocol of this trial.

## 5 Conclusion

In this study, we have confirmed that the passive elastic properties of the biceps brachii are consistent with the exponential model. The Young’s modulus–elbow angle curve of the biceps brachii changes in distinct ways depending on the muscle tone status. SWE can be used to quantify muscular stiffness during passive stretching as a new way of muscle tone evaluation, providing the possibility of a quantitative and mathematical model of muscle mechanical properties for the assessment of muscle tone in stroke patients.

## Data Availability

The raw data supporting the conclusion of this article will be made available by the authors, without undue reservation.
