# Supplementary material for: Quantitative assessment of biceps brachii muscle stiffness by using Young’s modulus–Angle curve during passive stretching in stroke patients
Source: Front Physiol. 2023 Mar 8;14:907337. doi: 10.3389/fphys.2023.907337 (PMC10030944; doi:10.3389/fphys.2023.907337)
Supplement: Supplementary file 1 [file Table1.DOCX]

Quantitative assessment of biceps brachii muscle stiffness by using Young’s modulus-angle curve during passive stretching in stroke patients

1. **Supplementary table 1. Influence of age on *E_X_* at different positions in control group.**

| Position | Youth (n=60) | Middle-aged (n=25) | Elderly (n=29) | χ^2^ value | *p* value |
| --- | --- | --- | --- | --- | --- |
| O: 81°, start point | 7.600 (6.925, 9.100) | 8.100 (6.500, 10.250) | 7.200 (6.500, 9.000) | 2.670 | 0.263 |
| A: 90°, 3/8 ROM | 8.250 (6.800, 9.875) | 8.300 (7.400, 10.350) | 8.000 (6.800, 8.800) | 1.828 | 0.401 |
| B: 108°, 1/2 ROM | 9.650 (8.300, 11.300) | 9.800 (8.300, 12.050) | 9.000 (7.800, 13.100) | 0.654 | 0.721 |
| C: 126°, 5/8 ROM | 11.250 (9.425, 13.075) | 11.700 (8.950, 17.450) | 10.700 (8.550, 19.050) | 0.478 | 0.787 |
| D: 144°, 3/4 ROM | 13.000 (10.975, 15.600) | 13.300 (10.250, 17.700) | 12.900 (10.350, 22.350) | 0.180 | 0.914 |
| E: 162°, 7/8 ROM | 15.450 (13.425, 19.075) | 14.400 (13.400, 21.250) | 16.200 (13.600, 24.650) | 1.134 | 0.567 |
| F: 180°, end point | 20.050 (16.900, 23.575) | 19.300 (15.250, 29.800) | 19.400 (16.750, 25.050) | 0.145 | 0.930 |

In the control group, the participants were divided into youth:<45 years; middle-aged: 45-60 years; and elderly: >60 years according to their age. Kruskal-Wallis H test showed that there was no statistically significant difference in Young's modulus at each position in different age groups. The Young’s modulus data in the table were shown as median (lower quartile, upper quartile) (kPa). Abbreviation: ROM: range of motion.

1. **Supplementary table 2. Influence of sex on *E_X_* at different positions in control group.**

| Position | Male (n=70) | Female (n=44) | Z value | *p* value |
| --- | --- | --- | --- | --- |
| O: 81°, start point | 8.050 (7.100, 9.400) | 7.100 (6.025, 8.575) | -2.857 | 0.004 |
| A: 90°, 3/8 ROM | 8.350 (7.275, 10.300) | 7.800 (6.525, 8.975) | -2.285 | 0.022 |
| B: 108°, 1/2 ROM | 9.750 (8.275, 11.925) | 9.600 (7.800, 11.300) | -0.818 | 0.413 |
| C: 126°, 5/8 ROM | 11.400 (8.975, 14.625) | 11.100 (9.350, 14.175) | -0.175 | 0.861 |
| D: 144°, 3/4 ROM | 13.000 (10.525, 18.825) | 13.050 (11.225, 15.800) | -0.451 | 0.652 |
| E: 162°, 7/8 ROM | 15.650 (13.300, 23.300) | 15.700 (13.600, 19.075) | -0.084 | 0.933 |
| F: 180°, end point | 20.200 (16.375, 27.675) | 19.800 (16.900, 23.300) | -0.384 | 0.701 |

At position of O-81° and A-90°, Young’s moduli were lower in female than that in male (p>0.05). The Young’s modulus data in the table were shown as median (lower quartile, upper quartile) (kPa). Abbreviation: ROM: range of motion.
